# Performance Enhancement of Solar Cell by Incorporating Bilayer RGO‐ITO Smart Conducting Antireflection Coating

**DOI:** 10.1002/gch2.201800109

**Published:** 2019-04-08

**Authors:** Anupam Nandi, Sukanta Dhar, Sanhita Majumdar, Hiranmay Saha, Syed Minhaz Hossain

**Affiliations:** ^1^ Centre of Excellence for Green Energy and Sensor Systems Indian Institute of Engineering Science and Technology (IIEST) Shibpur, Howrah 711103 West Bengal India; ^2^ National Institute of Technology Ravangla 737139 Sikkim India; ^3^ Department of Physics Indian Institute of Engineering Science and Technology (IIEST) Shibpur, Howrah 711103 West Bengal India

**Keywords:** bilayers, graphene, ITO, antireflection coatings, solar cells

## Abstract

Multilayered graphene deposited on a flat resistive surface has twofold benefits. Less electronic scattering reduces the sheet resistance of the combined bilayer and high photon scattering through the unavoidable wrinkles on the chemically synthesized graphene layer leads to decreased effective reflection. In this paper, wet‐chemically‐synthesized reduced graphene oxide (RGO) has been employed on the top of the indium‐doped tin‐oxide (ITO) layer. The ITO layer of optimized thickness has been deposited as an alternative antireflection coating (ARC) on a p/n junction based crystalline silicon solar cell with standard textured surface. Variation in spectral response has been studied experimentally for different thickness and surface coverage of RGO on ITO. The combined effect of reduced sheet resistance due to high surface conductivity and increased photon injection efficiency due to scattering from the wrinkles of RGO results in significant improvement in the performance of the solar cell. By employing optimum thickness of RGO, percentage enhancements of about 18% and 10%, respectively, in efficiency and short‐circuit current density have been achieved over the baseline cell structure. RGO also exhibits an additional benefit as a moisture repelling layer.

## 1 Introduction

In the area of nonconventional and renewable energy technology, solar cells have drawn enormous attention. Earlier, many researchers have contributed their footsteps and discussed their viewpoints in the sector of screen‐printed (p–n junction) textured silicon solar cell,^1^ amorphous silicon solar cell,^2^ Schottky Junction solar cell,^3^ dye‐sensitized solar cell,^4^ heterojunction with intrinsic thin‐layer (HIT) solar cell,^5^ and organic solar cell.^6^ A crystalline p–n junction solar cell has attracted the researcher much due to its higher end efficiency^7^ and durability^8^ among all. Amorphous silicon solar cell came in front row of research due to its low consumption of raw material.^9^ HIT solar cell has become a frontier research focus in solar cell regime due to its high end productivity and high efficiency.^10^ Solar cell technology deals with many crucial technical issues, viz., i) the junction formation, ii) electrical contact, iii) doping density, and most importantly, iv) the entrapment of photons.^11^, ^12^ In optical perspective the absorption of photons in the active layer produces the electron hole pair, which in turn produces the current after crossing the junction.^11^, ^12^ In order to enhance the absorption of photons, many researchers had adopted texturing the front surface of the silicon wafer to minimize the top surface reflection.^13^ Later on many dielectric materials with refractive index (RI) ≈ 1.6–2.1 have been incorporated as thin film window layer with a thickness of ≈70 to ≈90 nm on screen printed silicon solar cell to extract anti reflection^14^ along with passivation^12^ benefit. On the other hand, transparent conducting oxide (TCO), such as fluorine‐doped tin‐oxide (FTO),^15^ indium‐doped tin‐oxide (ITO),^16^, ^17^ and aluminum‐doped zinc‐oxide (AZO),^16^, ^17^ has been incorporated in the amorphous and the HIT solar cell^18^, ^19^ as electrode with a limitation in passivation. Thus, application of antireflection coating on c‐Si solar cell has been preferred than TCO.

2D monoatomic nanostructure, graphene, has attracted most due to its i) fascinating ballistic electron transport,^20^, ^21^ ii) high carrier mobility up to 200 000 cm^2^ V^−1^ s^−1^,^22^ and iii) nearly transparent (≈97.7%) nature in solar cell spectrum (200–1100 nm).^23^ Sheet resistance of overlayered (≈3–4 layers) RGO is 350 Ω □^−1^ with transmittance ≈93%^24^, ^25^ respectively in both visible and near‐infrared regions. Many researchers have shown the potential attributes of graphene as transparent conducting electrode material on screen printed textured silicon solar cell,^23^, ^26^ Schottky Junction solar cell,^27^ dye‐sensitized solar cell,^28^ and organic solar cell.^29^ A very limited study of RGO on crystalline silicon solar cell has been reported^23^, ^26^ and moreover the use of ITO in the same kind of solar cell structure is still an unexplored research regime. In this paper, all these remarkable properties of graphene stimulated us to develop a hybrid smart antireflection coating (ITO‐graphene composite layer), which can overcome the limitations of ITO by reducing the sheet resistance further and increases in the injected photon fraction.

In this paper, a basic structure of c‐Si solar cell (p–n junction) with metal electrodes on both sides has been fabricated, where ITO has been utilized as the photon trapping and carrier collecting layer on the c‐Si solar cell. RGO has been incorporated on top of the ITO layer for additional optical and electrical benefit. We have also reported the detailed evaluation of RGO coated/uncoated ITO based c‐Si solar cell. The improvement of the sheet resistance and photon trapping fraction has also been investigated after incorporation of RGO on ITO. The respective solar and spectral performances have been scrutinized by the current–voltage (*I*–*V*) study and optical (reflection, absorption, and transmittance) analysis, and validated by external quantum efficiency (EQE) measurements. Graphene has been synthesized by modified hummer's method.^30^, ^31^ Respective atomic force microscopic (AFM) study, Raman spectroscopic analysis, and field emission scanning electron microscopic (FESEM) analysis have been performed to confirm the formation of multilayered RGO film and its thickness. Optically suitable thickness of RGO layer has been optimized and realized through a step‐wise of comparative study. ITO has been deposited by radio frequency (RF) magnetron sputtering from ITO target. RGO exhibited an additional benefit as moisture protecting layer on solar cell leading to a durability enhancement.

## 2 Results and Discussion

Deposition of ITO thin film has been characterized by X‐ray diffraction (XRD) study and the respective signature planes <222>, <440> at 30° and 45.5° of 2θ axes confirm the formation of the ITO.^16^, ^17^ Surface morphology suggests the assembly of granular shaped ITO nanoparticles from the ITO thin layer. Detailed study has been discussed in Section 1 (Supporting Information). A careful experimental and mathematical data analysis is needed to optimize the optical thickness of the ITO that can be employed as TCO material on silicon solar cell for reproducible results. Optical property of ITO varies significantly with the variation in thickness of ITO layer, discussed in the Supporting Information. 80 nm thick ITO with transmittance of 87% and sheet resistance of 50 Ω □^−1^ (Supporting Information) has been established as the most suitable, hence used as the standard thickness for our further use in solar cell application. Formation of RGO can be confirmed by the change in intensity ratios of D and G band (*I*_D_/*I*_G_), which are 0.91 and 1.04 in GO and RGO.^20^, ^21^, ^23^, ^24^ Detailed has been discussed in 3 (Supporting Information).

### 2.1 Optimization of RGO Layer on Optimized ITO Layer (ITO_80)

In order to improve the quality of the ITO in terms of solar cell properties (Optical and electrical), RGO has been deposited on ITO substrate (ITO_80) to extract the further benefit. Due to nonavailability of substantial information of RGO–ITO composite TCO study from the previous research, we made a variation of thickness of RGO on previously optimized ITO_80 (80 nm thick ITO on experimental substrates), discussed in the Supporting Information. In this case we made a quantitative analysis of the benefit of photon injection and reduction in the sheet resistance of the RGO–ITO composite over the ITO_80 in a novel manner. We varied the thickness of coating of the RGO layer on ITO and recorded the respective optical data (Reflectance, absorbance, and transmittance) in the active solar region (Wavelength of 300–1100 nm) to realize the betterment.

**Figure 1:**
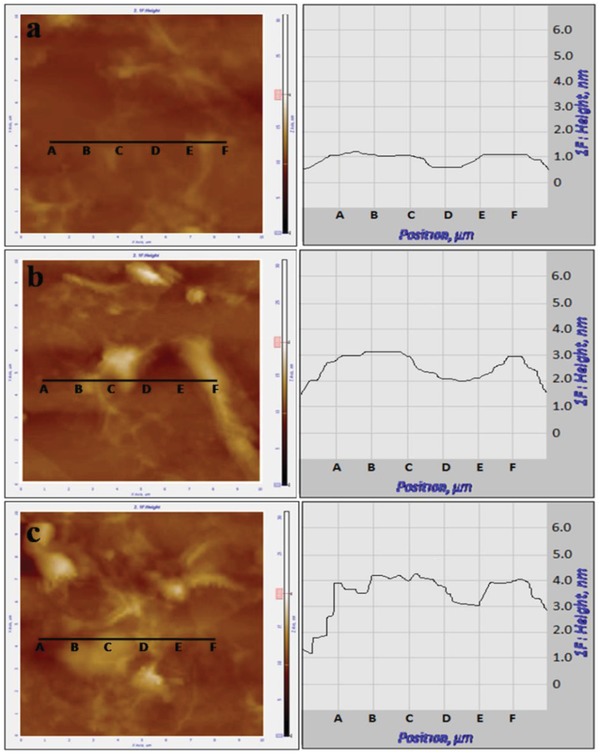
AFM microscopy image showing the thickness variation of RGO layer after spin coating RGO colloid of different densities. a) 15 µgm mL^−1^, b) 25 µgm mL^−1^, and c) 35 µgm mL^−1^ on polished silicon wafer.

**Figure 2:**
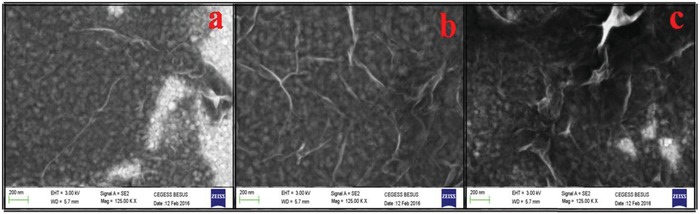
FESEM microscopy image of RGO layer after spin coating RGO colloid of different densities. a) 15 µgm mL^−1^, b) 25 µgm mL^−1^, and c) 35 µgm mL^−1^ on 80 nm thick ITO.

Respective photon fraction has been calculated to optimize the density of RGO, which produces RGO layer of ideal thickness leading to the maximum surface coverage for the enhancement of optical property over ITO_80. Colloidal suspensions of RGO in IPA of different densities (15, 25, and 35 µgm mL^−1^) have been spin coated on the polished silicon wafer followed by the room temperature drying for the thickness measurement of the RGO layer by the AFM tool. Respective AFM surface morphology and height profile (**Figure** **1**) of the deposited RGO layer for different densities (15, 25, and 35 µgm mL^−1^) suggest that the average thickness of the RGO layer increases with the increment of the density of the RGO colloid. It is evident that the average thicknesses of the RGO layer for the different RGO colloids (15, 25, and 35 µgm mL^−1^) are ≈1 nm (Figure 1a), ≈2.2 nm (Figure 1b), and ≈3.1 nm (Figure 1c), respectively, suggesting the formation of 0–1 layers, 2–3 layers, and 4–5 layers, respectively. Colloidal RGO of variable densities (15, 25, and 35 µgm mL^−1^) spin coated on ITO_80 coated glass substrate in identical condition for transmittance, absorbance, and FESEM study to investigate the surface coverage of RGO (**Figure** **2**). It is evident that surface coverage of RGO increases with the increment of density of RGO, when coated on the ITO_80_glass substrate. The RGO colloid with density 15 µgm mL^−1^ shows poor coverage of RGO without any significant overlayer and agglomerations of RGO (Figure 2a).

**Figure 3:**
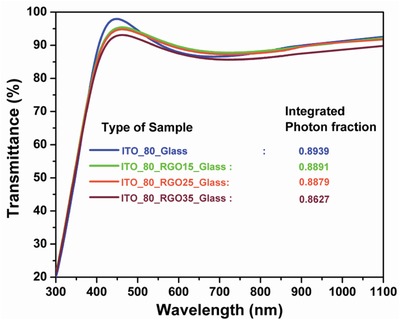
Transmittance curve of different thicknesses of RGO on 80‐nm‐thick ITO coating on glass substrate.

Though the best coverage can be achieved by coating 35 µgm mL^−1^ RGO colloid but shows highest agglomeration in the structure (Figure 2c), which is also not desirable for solar cell as overlayer creates hindrance to the photons to get inside the active layer. A tradeoff structure is needed to be achieved where the surface coverage is in the acceptable range without any significant over layer or agglomerations. The RGO colloid with density 25 µgm mL^−1^ has been established as the most suitable (Figure 2b) as it holds both the aforesaid criteria. ITO_80 and other respective RGO coated ITO_80 glass substrates, viz., ITO_80_RGO15_glass (RGO with 15 µgm mL^−1^ density coated on 80 nm thick ITO), ITO_80_RGO25_glass (RGO with 25 µgm mL^−1^ density coated on 80 nm thick ITO), and ITO_80_RGO35_glass (RGO with 35 µgm mL^−1^ density coated on 80 nm thick ITO) have been prepared to compare the transmission spectra within the regime of 300–1100 nm. The calculated integrated transmission fraction (Equation S5, Supporting Information)^23^, ^32^ of ITO_80_glass is 0.8939, which has been considered as the reference substrates to perceive the benefit of RGO of different thickness on ITO_80 (**Figure** **3**).

**Figure 4:**
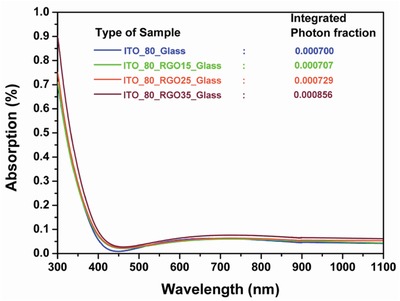
Absorbance curve of different thicknesses of RGO on 80 nm thick ITO coating on glass substrate.

Respective transmission reduces linearly with the increment of the density of the RGO colloid, when coated on ITO_80 glass substrate. The integrated transmittance fractions through ITO_80_RGO15_glass, ITO_80_RGO25_glass, and ITO_80_RGO35_glass are 0.8891, 0.8879, and 0.8727, respectively (**Table** **1**). The reduction of transmitted photons during the time of photon travel is highly dependent on the thickness of the RGO layer as the base material is identical for all the measured samples, i.e., ITO_ 80_glass, validated by the absorption data of the respective RGO–ITO coating samples. From the absorption graph (**Figure** **4**) it can be apprehend that the gradual increment of the RGO density shows increment in absorption as the thickness of RGO increases. Photons are absorbed in the RGO layer as light travels greater path through the RGO with the increment of thickness of RGO at the cost of reduction in effective transmittance. The absorbed photon fraction increases from 7.07 × 10^−4^ to 8.56 × 10^−4^ with the increment of the density of the RGO colloid from 15 to 35 µgm mL^−1^ (Equation S4, Supporting Information),^23^, ^32^ tabulated in Table 1.

**Table 1:** Calculation of photon fraction from reflection, absorption, and transmittance of RGO of different thicknesses on 80‐nm‐thick ITO

**Total photons(Global) 2.1034 × 10^17^ cm^−2^ s^−1^**	**Type of sample**	**On Wafer(substrate)**		**On Glass (substrate)**		**On Glass (Substrate)**		
		**TRP**	**RPF**	**TAP**	**APF**	**TTP**	**TPF**	**IPF**
	ITO_80	5.79 × 10^15^	0.027	1.4716 × 10^14^	7E‐4	1.88 × 10^17^	0.8939	0.8693
	ITO_80_RGO15	4.98 × 10^16^	0.023	1.4871 × 10^14^	7.07E‐4	1.87 × 10^17^	0.8891	0.8680
	ITO_80_RGO25	4.33 × 10^15^	0.019	1.5333 × 10^14^	7.29E‐4	1.86 × 10^17^	0.8879	0.8705
	ITO_80_RGO35	3.84 × 10^15^	0.018	1.8005 × 10^14^	8.56E‐4	1.83 × 10^17^	0.8727	0.8563

TP: total photons in AM1.5(Global) from 300 to 1100 nm, cm^−2^ s^−1^; TRP: total reflected photons from the top surface from wavelength 300 to 1100 nm, cm^−2^ s^−1^; RPF: reflected photons fraction (TRP/TP), no unit; TAP: total absorbed photons in ITO, cm^−2^ s^−1^; APF: absorbed photon fraction (TAP/TP), no unit; TTP: total transmitted photons through ITO layer from 300 to 1100 nm wavelength, cm^−2^ s^−1^; TPF: transmitted photons fraction through ITO layer (TTP/TP), no unit; ITPF: injected photon fraction {TPF × (1 − RPF)}, no unit.

**Figure 5:**
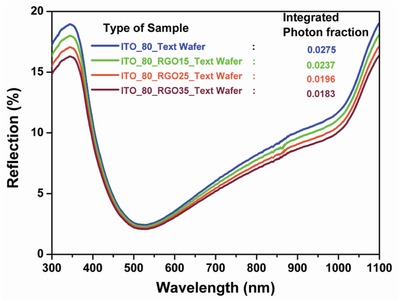
Reflection curve of different thicknesses of RGO on 80 nm thick ITO coating on textured silicon wafer.

In order to quantify the reflection benefit due to incorporation of RGO (various thicknesses) on optimized ITO layer (80nm) with textured wafer as base, has been compared with ITO_80 on text wafer, shown in **Figure** **5**. Respective reflected photon count and the integrated photon fraction have been calculated (Equation S3, Supporting Information)^23^, ^32^ and tabulated in Table 1.

The reflectance curve of ITO_80_RGO15_wafer (with 15 μgm mL^−1^) shows an insignificant downshift w.r.t. ITO_80_wafer. With the increment of density of RGO or in the other ways with the increment of thickness of the RGO layer, reflection from the top surface decreases accordingly. The presence of wrinkles in the RGO layer(FESEM microscopy image in Figure 2) enables the incident light to get scattered at the surface.^33^ Reduction of reflection with the increment of the RGO layer thickness does not at all infer that higher number of photons were harvested, as the corresponding transmittance graph (Figure 3) reflects a significant loss caused due to more absorption, supported by the absorption graph (Figure 4). In order to calculate the exact photon fraction injected to the solar cell, we have incorporate the entire reflected, transmitted, and absorbed photon fraction under consideration (Equation S6, Supporting Information).^23^, ^32^ It is observed from Table 1 that the best injected photon fraction can be achieved through ITO_80_RGO25, which is 0.8705. Whereas the calculated injected photon fractions for ITO_80, ITO_80_RGO15, and ITO_80_RGO35 are 0.0893, 0.8680, and 0.8563.

It is evident from the respective optical studies (transmittance, absorbance, and reflectance) that the RGO colloid with density 25 µgm mL^−1^ has been established as the most suitable optical layer on the ITO of thickness 80 nm. RGO of aforesaid densities (15, 25, and 35 µgm mL^−1^) has been coated on in‐house fabricated ITO_80_cell (baseline) in order to validate the optical optimization of thickness of the RGO layer (density of the RGO colloid) through enhancement of solar performance over the baseline solar cell. It has also been investigated that whether the application of RGO can trim down the absorption loss along with the decrease in sheet resistance, so that the carriers can be collected from the solar cell to the electrode with greater ease. Three types of solar cell with RGO–ITO hybrid coating (ITO_80_RGO15_cell, ITO_80_RGO25_cell and ITO_80_RGO35_cell) have been fabricated in order to check solar performance after incorporation of RGO on ITO_80 coated solar cell.

### 2.2 Solar Cell Performance Study

**Figure 6:**
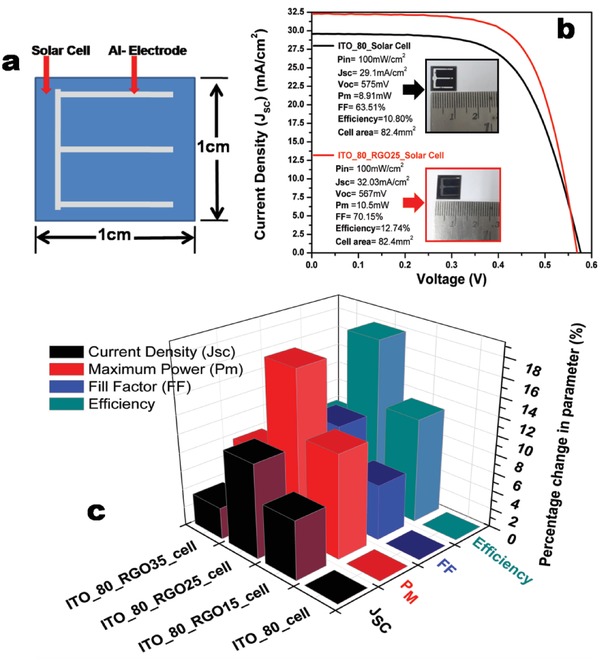
a) Schematic representation of solar cell device. b) Solar cell *I*–*V* graph under AM1.5. c) Bar diagram representation of percentage performance enhancement of RGO (Different thickness) coated solar cell over ITO_80_cell.

The *I*–*V* measurement of ITO_80_cell (80 nm thick ITO on solar cell), ITO_80_RGO15_cell (80 nm thick ITO coating on solar cell with coating of RGO of density 15 µgm mL^−1^ on ITO), ITO_80_RGO25_cell (80 nm thick ITO coating on solar cell with coating of RGO of density 25 µgm mL^−1^ on ITO), and ITO_80_RGO35_cell (80 nm thick ITO coating on solar cell with coating of RGO of density 35 µgm mL^−1^ on ITO) of area 1 cm^2^ has been carried out in order to realize the effect of RGO on baseline solar cell (ITO_80_cell). As the short‐circuit current (*I*_sc_) majorly depends upon the illuminated area of the solar cell, thermally evaporated aluminum electrode occupies a significant area of 0.176 cm^2^ (**Figure** **6**a) on the cell surface, which hinders the incident photons to pass through it during measurement under AM1.5; hence, the active illuminated area is considered to be 0.824 cm^2^. The results were analyzed in terms of i) open‐circuit voltage (*V*_oc_), ii) short‐circuit current density (*J*_sc_), iii) maximum power output (*P*_m_), iv) fill factor (FF), and v) efficiency (η). Further estimation of other intrinsic important factors such as i) series resistance (*R*_S_), ii) shunt resistance (*R*_Sh_), iii) reverse saturation current density (*J*_01_), and iv) diffusion current density (*J*_02_) were obtained by fitting respective *J*–*V* curve in solar cell two‐diode model matlab programming tool. From the solar performance data (**Table** **2**), it can be realized that with the increment of the thickness of the RGO layer (increasing the density of the RGO colloid), the overall performance has been affected significantly. ITO_80_cell shows *J*_sc_ of 29.1 mA cm^−2^ with 63.51% FF and maximum power of 8.91 mW. In the due course of travel of photogenerated carriers to the electrode through 80 nm thick ITO, a fraction of holes recombines in the ITO, which can be recovered tactfully.

**Table 2:** Solar performance statistics of ITO and different RGO–ITO layer‐based solar cell

Type of Solar Cell (SC)	Avg. Thickness of RGO layer	*V*_oc_ [mV]	*J*_sc_ [mA cm^−2^]	*P*_m_ [mW]	FF [%]	*ɳ* [%]
ITO_80_cell	0	575	29.1	8.9	63.51	10.80
ITO_80_RGO15_cell	≈1 nm	577	30.9	9.9	67.29	12.01
ITO_80_RGO25_cell	≈2.2 nm	567	32.0	10.5	70.15	12.74
ITO_80_RGO35_cell	≈3.1 nm	574	30.0	9.6	66.86	11.65

In order to cope with the loss fraction of the carriers (ITO_80), we enhanced the optical and electrical benefit of ITO by spin coating RGO on ITO_80_cell, which instigated us to search the suitable thickness of RGO by varying the density of the RGO. From Table 2, it is evident that the solar performance has been elevated, attributing a significant photon trapping due to the coating of RGO on ITO. Maximum photon injection through ITO_80_RGO25 causes maximum (Table 2) electron hole pair generation, which is significantly higher than the baseline solar cell and also claims to be the highest among the measured other solar cell, shown in Figure 6b. Noteworthy percentage enhancement of *J*_sc_ (10.06%), *P*_m_ (17.84), FF (10.45%), and efficiency (17.96%) has been achieved over the ITO_80_cell (**Table** **3**).

**Table 3:** Percentage enhancement of solar performance statistics of RGO layer of different thickness coated on 80‐nm‐thick ITO layer based solar cell over without RGO‐coated 80‐nm‐thick ITO‐based solar cell

	Percentage enhancement of *J*_sc_ [%]	Percentage enhancement of *P*_m_ [%]	Percentage enhancement of FF [%]	Percentage enhancement of efficiency (*ɳ*) [%]
ITO_80_cell	0	0	0	0
ITO_80_RGO15_cell	6.32	11.11	5.95	11.2
ITO_80_RGO25_cell	10.06	17.84	10.45	17.96
ITO_80_RGO35_cell	3.40	7.74	5.27	7.87

The enhancement in solar response not only accounts for the optical benefit of RGO–ITO hybrid film but also due to possible benefit of reduction in sheet resistance. Though the photon injection fraction through ITO_80 film is higher than ITO_80_RGO15, but the solar response of ITO_80_RGO15_cell is better than ITO_80_cell because of the sheet resistance reduction. *J*_sc_, FF, and efficiency of ITO_80_RGO15_cell show a percentage enhancement of 6.32%, 5.95%, and 11.2% in *J*_sc_, *P*_m_, and efficiency over ITO_80_cell (Table 3), shown in Figure 6c. The photogenerated carriers prefer to reach to the electrode through continuous RGO network instead of the ITO layer as the conductivity of the graphene is higher than ITO. The enhancement in solar cell performance of ITO_80_RGO35_cell is poorer than ITO_80_RGO15_cell and ITO_80_RGO25_cell. The variation of the solar cell response noticeably varies with the incorporation of the RGO of different thicknesses (varying the density of the RGO colloid), which is outlined to compare in perceptible manner in Figure 6c and the compared data have been tabulated in Table 3. As per the solar performance table and the photon calculation data, it is persuasive that the hybrid coating of ITO and RGO (25 µgm mL^−1^), viz. ITO_80_RGO25, is proved to be the optimized and optically potent window layer, obtained from respective optical and solar cell studies.

Further analysis, viz., series resistance, *J*_01_ (reverse saturation current density), and *J*_02_ (diffusion current density), has been carried out by fitting the respective characteristics curves in the two‐diode model in the matlab programming tool. Series resistance (*R*_S_) is the summation of the entire resistance experienced by carriers during the course of travel from the top of the n‐type active silicon layer to the point of collection. In due course of this travel, resistances are offered by i) ITO along the thickness, ii) sheet resistance of the top surface of the solar cell to the aluminum electrode, and iii) the resistances of the thermally deposited metal electrode. *R*_S_ of a solar cell determines the probability of collecting carriers at the terminal. It is also observable that with the increment of thickness of the RGO layer on top of the ITO reduces the equivalent sheet resistance of the ITO–RGO hybrid window layer gradually. Though the sheet resistance of ITO_80_RGO35 (33.1 Ω □^−1^) is the lowest among all other RGO–ITO hybrid layers, it does not always confirm the best solar performance because it is also evident that the injected photon fraction is the least for ITO_80_RGO35, shown in Table 1. Both the optical and electrical parameters, viz., transparency and sheet resistance, were accounted at the same time in order to develop a solar potent TCO. ITO_80_RGO25 claims to be the best in our study as the fraction of injected photon is highest and the sheet resistance is also comparable to ITO_80_RGO35, which has been supported further by the relevant *J*–*V* analysis. It is also found from **Table** **4** that *J*_01_ of ITO_80_RGO25_cell and ITO_80_RGO35_cell are almost identical (4.188 × 10^−14^ mA cm^−2^) and also lower than that of ITO_80_cell (*J*_01_ = 1.76 × 10^−13^ mA cm^−2^) and ITO_80_RGO15_cell (*J*_01_ = 2.095 × 10^−13^ mA cm^−2^).

**Table 4:** Derived cell parameters of 80 nm thick ITO coated silicon solar cells along with RGO coating of different thicknesses

Type of Solar Cell (SC)	*J*_01_ [mA cm^−2^]	*J*_02_ [mA cm^−2^]	*R*_S_ [Ω cm^2^]	Sheet resistance
ITO_80_cell	1.76 × 10^−13^	4.93 × 10^−11^	0.02	50 Ω □^−1^
ITO_80_RGO15_cell	2.10 × 10^−13^	1.92 × 10^−11^	0.009	45.4 Ω □^−1^
ITO_80_RGO25_cell	4.18 × 10^−14^	3.16 × 10^−12^	0.005	39.7 Ω □^−1^
ITO_80_RGO35_cell	4.18 × 10^−14^	3.15 × 10^−12^	0.001	33.1 Ω □^−1^

The coating of RGO on ITO_80_cell produces stronger electric field (between n‐doped Si region and p‐doped Si region) due to the separation of intrinsic charges in each of the active solar cell layer. Electron‐rich RGO layer produces field in between ITO and RGO, resulting an accumulation of more electrons at the base of the ITO (in contact with n‐type silicon layer) that helps to produce a strong electric field in between ITO (with electrons at base of ITO layer) and n‐type silicon layer (with holes near the top of n‐type silicon layer). This surface activated electric field on n‐type silicon layer produces higher electric field in between the n‐type and p‐type silicon layers due to the coating of the RGO layer. This cascading arrangement of separated charges in the hierarchy of the solar cell produces stronger electric filed, which in turn incorporates the lower *J*_01_ through the barrier. From Table 4, it is evident that *J*_01_ decreases (strength enhancement of electric field) with the increment of the RGO layer thickness and saturated after second RGO coating, attributing no further improvement is attainable in terms of electric field strength. This enhanced electric field hinders the diffusion of electrons (from n‐type to p‐type) and holes (from p‐type to n‐type), attributing a decrement in diffusion current density (*J*_02_). With the increment of the RGO coating on baseline solar cell, a gradual reduction of *J*_02_ (4.93 × 10^−11^ to 3.15 × 10^−12^ mA cm^−2^) can also be observed, shown in Table 4.

As the diffusion of carriers is highly dependent upon the electric field (in between n‐type and p‐type Si layer), the diffusion current density of ITO_80_RGO35_cell is almost identical with that of ITO_80_RGO25_cell, attributing no further improvement can be achieved. From the detailed analysis of *R*_S_, *J*_01_, and *J*_02_, it is found that ITO_80_RGO25_cell claimed to be the appropriate in terms of potential photon compilation and also validated from the respective solar response graph and *I*–*V* characteristics.

**Figure 7:**
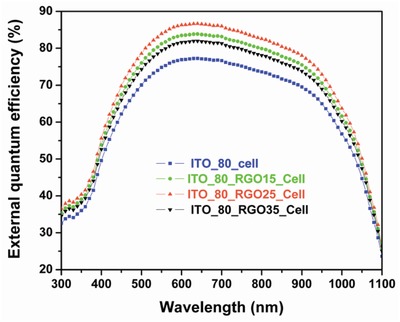
External quantum efficiency (EQE) plot of baseline solar cell (ITO_80_cell) and different RGO‐coated baseline solar cell.

The enhancement of EQE (**Figure** **7**) of ITO_80_RGO25_cell is noteworthy than that of ITO_80_cell and other RGO coated solar cells (ITO_80_RGO15_cell and ITO_80_RGO35_cell). From Figure 7, it can be suggested that the enhancement has been achieved within the full range of solar spectrum (300–1100 nm) due to the application of RGO over the baseline solar cell (ITO_80_cell). It is comprehensive that, with the increment of the density of the RGO (thickness of the RGO layer), the external quantum efficiency is enhanced by a notable fraction.

**Figure 8:**
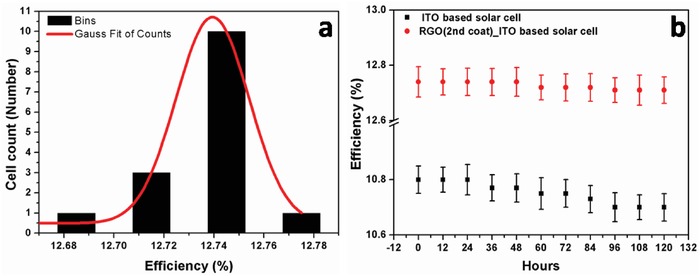
a) Statistical presentation of solar performance data graph of ITO_80_RGO25_cell and b) photoinduced degradation study of ITO_80_RGO25_cell with error bar.

Enhancement of EQE can be noticed till ITO_80_RGO25_cell but reduces for overlayered RGO coated ITO_80_cell, i.e., ITO_80_RGO35_cell, as the formation of overlayer and/or agglomerations hinders the photons to get inside the active layer of solar cell (Figure 7). In order to check the repeatability of the fabrication of device, 15 numbers of ITO_80_cell cells have been fabricated in the same batch. *I*–*V* measurements of all the 15 solar cells are characterized under AM1.5G and are almost identical to the data of ITO_80_cell, mentioned in Table 3. RGO with optimized density (25 µgm mL^−1^ density) has been coated on the top surface of all the fabricated 15 solar cells by identical deposition technique in order to develop ITO_80_RGO25_cell. Solar performances of the 15 ITO_80_RGO25_cell were measured under AM1.5G and conversion efficiency has been statistically tabulated with a Gaussian fitting in **Figure** **8**a. It is noticeable that the efficiency (≈12.74%) of ten cells was found to be almost identical to the previously mentioned for ITO_80_RGO25_cell, supporting the fabrication repeatability of ITO_80_RGO25_cell with 12.74% efficiency. Another statistical data have been represented, where ITO_80_cell and ITO_80_RGO25_cell were continuously illuminated under AM1.5G for 120 h for photoinduced degradation study (Figure 8b), showing a steady output throughout the illuminated condition. Besides many potential attributes, RGO can be used as the protecting layer on suitable device structure. In this work, RGO has been used as coating for weather insulation sheet on solar cell as it hinders the areal moisture^34^ and enhances the durability of the fabricated cell.

**Figure 9:**
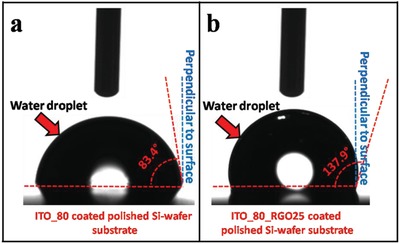
Contact angle measurement study of water droplet on a) ITO_80‐coated polished Si‐wafer substrate and b) ITO_80_RGO25 coated polished Si‐wafer substrate.

In our study, ITO_80_RGO25_cell has been established to be the best photoconversion device with highest efficiency. In order to establish RGO coating as moisture insulation on ITO, contact angle measurement test using Drop Shape Analyzer (KRUSS GmbH—DSA25) instrument has been performed on ITO_80 and ITO_80_RGO25_cell. It is noticed that the contact angles of water droplet on the ITO_80 coated polished silicon surface and ITO_80_RGO25 coated polished silicon substrate are 83.4° (**Figure** **9**a) and 137.9° (Figure 9b), respectively. It can be apprehended that the absorption of areal water molecule can be reduced by a considerable fraction by coating RGO on the solar cell structure, making an impenetrable inert layer on the solar cell structure.

## 3 Conclusion

In this report, ITO has been deposited by RF sputtering technique and characterized by XRD and FESEM study. Detailed optical analysis has been performed to establish the optimized thickness of ITO (80 nm), validated by optical data and graph (reflectance, absorption and transmittance). ITO layer of thickness of 80 nm has been used on shallow n‐doped (grown on p‐type wafer) silicon wafer as the ARC on top of the solar cell hierarchy (considered as baseline solar cell) with an injected photon fraction of 0.8693. *J*_sc_, FF, and efficiency of baseline solar cell have been recorded as 29.1 mA cm^−2^, 63.51%, and 10.8%, respectively, under AM1.5G. In urge to improve the solar performance (in terms of injected photon fraction and sheet resistance) of baseline solar cell (ITO_80_cell), RGO has been applied on top of the ITO of the baseline solar cell. RGO has been synthesized by hummer's method and characterized by Raman spectroscopy to establish the formation. Density of the RGO colloid in IPA has been varied as 15, 25, and 35 µgm mL^−1^ and deposited on polished silicon wafer and 80 nm thick ITO layer, in order to investigate the morphology and layer pattern from respective AFM and FESEM microscopy images. In order to attain the optimized thickness of RGO on optimized ITO layer (80 nm), respective optical (reflectance transmittance and absorbance) graphs and morphological study (AFM and FESEM) have been considered for the comprehensive justification and detailed analytical study. Through a proper analytical and experimental approach, it is found that the RGO colloid of density 15 µgm mL^−1^ enable to form RGO layer of thickness ≈2.0–≈3.0 nm on baseline solar cell after coating and drying, which is able to inject 0.8705 fraction of photons form the solar spectrum AM1.5G and claimed to be the best than other densities RGO. 2.52 × 10^15^ cm^−2^ s^−1^ numbers of photons are injected more in the solar cell device due to the coating of RGO of 15 µgm mL^−1^ on ITO_80_cell than that of uncoated ITO_80_cell. *J*_sc_, FF, and efficiency of ITO_80_RGO25_cell have been recorded as 32.03 mA cm^−2^, 70.15%, and 12.74% respectively under AM1.5G, showing a noteworthy percentage enhancement of *J*_sc_ (10.06%), FF (10.45%), and efficiency (17.96%) over the baseline solar cell, validated from the external quantum efficiency study. RGO also act as protecting layer on the solar cell structure from areal moisture.

## 4 Experimental Section

DC magnetron sputtering unit has been used to grow ITO thin film of controlled thickness on the desired substrate. RGO has been synthesized by the reduction of graphene oxide following the well‐established modified hummer's method. p‐Type boron‐doped crystalline silicon (p‐type <100> CZ) wafer (thickness: 180 µm and resistivity 1–2 ohm cm) has been procured and used as the substrate material of the solar cell.

*Development of ITO*: ITO films were prepared on desired substrates like glass, polished wafers, and n‐side of the diffused n–p wafer by DC magnetron sputtering system with power density of 1 W cm^−2^ at an elevated temperature (150 °C). A sintered target of In_2_O_3_ and SnO_2_ in a weight proportion of 9:1 was used for the ITO deposition with a controlled deposition rate of 5 nm min^−1^. The Ar and oxygen flow ratio was maintained at 100:1 with a base vacuum of 1 5 × 10^−6^ mBar.

*Reduced Graphene Oxide Synthesis*: Reduced graphene oxide was synthesized by wet chemical technique, where graphite powder (Laboratory Burgoyne Reagents) of average flake size of 25 µm was exfoliated to thinner flakes by low‐temperature oxidation process followed by reduction process, reported elsewhere.^23^

*Material Characterization Measurement of ITO and RGO*: In order to establish the formation of ITO thin film of different thickness, X‐ray diffraction (RIGAKU Ultima IV, Cu as laser source with wavelength of ≈1.5418 Å) study was performed. Formation of RGO was validated by Raman spectroscopy (RenishawinVia with a laser line of 514 nm).

*Preparation of ITO Samples*: ITO of thicknesses 40, 60, 80, and 100 nm was deposited on substrates like cleaned glass, textured wafer, and cleaned polished silicon for optical measurements and henceforth named as ITO_40, ITO_60, ITO_80, and ITO_100. ITO_80 was established as the most suitable thickness of ITO on solar cell, through which photon could be trapped most. In‐depth optical analysis of ITO coated samples is discussed in the Supporting Information.

**Figure 10:**
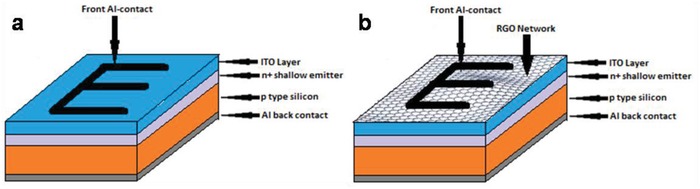
Basic solar cell structure of a) ITO_80 based solar cell, b) RGO‐coated ITO_80 based solar cell. “E” shaped aluminum contact on both types of cell is fabricated by thermal evaporation technique with proper mask.

*ITO‐Based Solar Cell Fabrication*: After the prerequisite chemical treatment p‐type boron‐doped crystalline silicon (p‐type < 100> CZ) wafer were treated for the formation of n^+^ layer on the wafer by a conventional POCl_3_ diffusion in diffusion furnace (SVCS‐ SVFUR‐AH3). Aluminum paste was then screen printed p‐side of the wafer followed by a cofire technique and considered as rear contact of the device.^2^, ^5^, ^8^, ^9^, ^10^, ^12^ ITO of thickness 80 nm (ITO_80) was deposited on the n^+^ side of the wafer by RF magnetron sputtering. Then electrical contact was made on the ITO coated side by depositing patterned aluminum fingers by thermal deposition using “E” mask (shown in **Figure** **10**a) and considered as front side of the fabricated cell. The reason behind considering the ITO of thickness 80 nm has been discussed in the Supporting Information, henceforth the basic solar cell structure with 80 nm thick ITO layer has been named as ITO_80_cell (Figure 10a).

*Coating of RGO on ITO_80 Substrates (Wafer, Glass, and Solar Cell)*: In order to attain the best coverage of RGO of suitable thickness, the density of the RGO colloid in IPA medium was varied as 15, 25, and 35 µgm mL^−1^. The density of the RGO colloid was established in the previous work.^23^ 5 mL of each of the colloid was spin coated on ITO coated (80 nm) substrates (Wafer, glass, and solar cell of area 1 cm^2^) followed by room temperature vacuum drying. ITO substrates coated with RGO colloidal suspension of densities of 15, 25, and 35 µgm mL^−1^ were henceforth named as ITO_80_RGO15, ITO_80_RGO25, and ITO_80_RGO35. Glass substrate and textured silicon wafer were considered as the reference substrate for transmission and refection measurement and solar performance measurement was carried out on ITO_80_cell in dark and under light (AM1.5). RGO coated ITO_80_cell structure has been demonstrated in Figure 10b.

*Optical/Electrical Characterization of Fabricated Substrate and Solar Cells*: The thickness and coverage of graphene layer of various densities on polished silicon substrates were investigated by AFM (NT‐MDT: Model No. 50BM‐4) and FESEM (ZEISS: Sl. No. SIGMA‐02‐87) microscopy images, respectively. Percentage loss of respective transmission and absorption of ITO_80_RGO15, ITO_80_RGO25, and ITO_80_RGO35 on glass substrate were compared and investigated with the bare glass substrate and ITO_80_glass. Reflection data from the surfaces of ITO_80_RGO15, ITO_80_RGO25, and ITO_80_RGO35 coated textured silicon wafer were recorded in Bentham PVE 300 system and compared with the bare textured wafer and ITO_80 coated textured wafer to estimate the reflection benefit. Dark and light (AM1.5) current–voltage (*I*–*V*) characteristic curves of RGO coated (various densities) and uncoated solar cells were tested using the *I*–*V* tool (PET: Photo Emission Tech. Inc.; Model 60623). EQEs were measured using Bentham PVE 300.

## Conflict of Interest

The authors declare no conflict of interest.

## Supporting information

Supplementary
