# Supplementary material for: Performance Enhancement of Solar Cell by Incorporating Bilayer RGO‐ITO Smart Conducting Antireflection Coating
Source: Glob Chall. 2019 Apr 8;3(8):1800109. doi: 10.1002/gch2.201800109 (PMC6686172; doi:10.1002/gch2.201800109)
Supplement: Supplementary file 1 — Supplementary [file GCH2-3-1800109-s001.pdf]

Copyright WILEY-VCH Verlag GmbH & Co. KGaA, 69469 Weinheim, Germany, 2019.

Supporting Information

for *Global Challenges,* DOI: 10.1002/gch2.201800109

Performance Enhancement of Solar Cell by Incorporating Bilayer RGO-ITO Smart Conducting Antireflection Coating

*Anupam Nandi, Sukanta Dhar, Sanhita Majumdar, Hiranmay Saha, and Syed Minhaz Hossain**

Electronic Supplementary Information

Performance enhancement of solar cell by incorporating bilayer RGO-ITO smart conducting antireflection coating

AnupamNandi[a], SukantaDhar[a], SanhitaMajumdar[a], HiranmaySaha[a]and Syed Minhaz Hossain[c*]

[a]*Centre of Excellence for Green Energy and Sensor Systems,*
Indian Institute of Engineering Science and Technology (IIEST),
Shibpur, Howrah 711103, West Bengal, India

[b]*Department of Electronics and Communication Engineering, National Institute of Technology, Sikkim, Ravangla, India - 737139*

[c]*Department of Physics*
Indian Institute of Engineering Science and Technology (IIEST),
Shibpur, Howrah 711103, West Bengal, India

***Email: shminhaz@physics.iiests.ac.in**

1. ITO deposition and Characterization

ITO of thicknesses 40nm, 60nm, 80nm and 100nm have been sputtered on glass and silicon wafer substrate of area 1cm^2. The said thicknesses were checked by the surface profilometer (DEKTAK-XT, Bruker). FESEM micrograph (Figure 1.a) of the sputtered ITO thin film of thickness 100nm on polished silicon wafer reveals a deposition of tiny random granular particle layer. Surfaces of all other ITO thin films of thicknesses 40nm, 60nm and 80nm exhibited same kind of granular deposition. From the X-ray diffraction all as-deposited ITO films contain the possible crystalline phases confirming the deposition of Sn doped In_2O_3 and well matched with JCPDS-ICDD No. 05-0848 (cubic bixbyite structure). With the increment of thickness, peak intensity increases, suggesting that the sputtered ITO attains the crystalline orientation accordingly. The two signature diffraction peaks corresponding to orientation along (222) and (400) plane are the strongest[1], shown in figure 1b. Due to less oxygen flow rate the growth occurs under Indium rich conditions, which lead to the preferential (<100>) texture. The gradual increment of the peak intensity corresponds to in 440 planes, which authenticates the

introduction of in-plane and inter-planner crystal defect in the deposited ITO layer, which supports the formation of desirable 222 plane, potential as TCO material. All X-Ray diffraction data suggest that the 222 oriented phases is the dominating phases than 400 oriented planes. The higher time oxygen exposure during the growth of 80nm and 100nm ITO layer is a possible reason for the presence of the two peaks suggesting planes 211, 024 and 136. However, we have no clear understanding about the preferential growth of these peaks at present [1, 2].

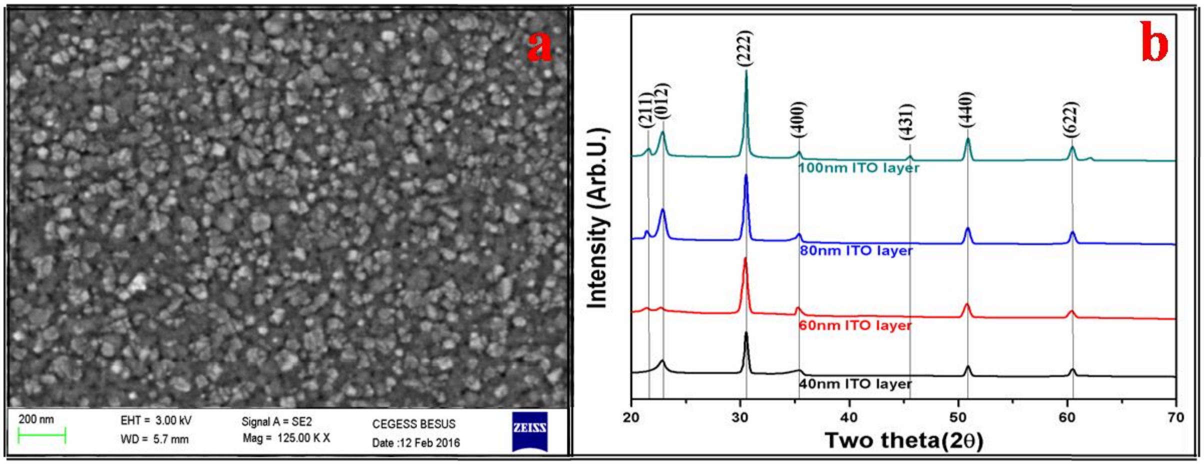

Figure 1: X-Ray diffraction peak of ITO thin film of thicknesses of 40nm, 60nm, 80nm and 100nm deposited on glass plate.

2. Optimization of ITO layer

A part of the light trapping structure includes lower reflection and absorption loss at the front layer of the device. The reflection takes place because of difference in refractive index between two layers. Refractive index (RI) of c-Si is 3.88 with that of air is 1 [3]. Thus, TCO film having refractive index somewhere around ~2 may show better optical transmission in accordance with equation 1 [4]. The quarter of the wavelength thickness of the ARC layer produces destructive interference causing least reflection overall according to the equation 2 [4].

Selection of ARC is so important, that effect of interference on the coating causes the wave reflected from the top surface of anti-reflection coating to be out of phase with the wave reflected from the semiconductor surfaces. These out-of-phase reflected waves destructively interfere with one another, resulting in zero net reflected energy.

From the equation 1, n_1 is the RI of the intermediate ARC layer i.e. ITO (in our case) between air with refractive index 1 (n_0) and silicon layer with refractive index 3.88 (n_2). The calculated RI

from equation 1 is 1.975, which is almost matched with the RI of ITO [5, 6] material as ARC in between air and silicon to harvest maximum nos. of photons. Thus parasitic reflection losses due to mismatch in RI can be minimized by incorporating ARC following the condition stated in equation 1. Lowest reflection from the surface of the ITO layer can be achieved if the thickness of the ARC should be quarter of the mid visible wavelength, required to be placed in between [4]. Solving equation 2, the lowest reflection will be obtained using ~80nm thick ITO.

$$n_1 = \sqrt{(n_0 \times n_2)} \quad (1)$$

$$d = {}^{\lambda}\!/_{(n \times 4)} \quad (2)$$

ITO films are highly degenerate n-type semiconductors and have low electrical resistivity (10^{-4} Ω-cm) along with high carrier concentration. Furthermore, ITO has a wide band-gap (Eg ≈ 3.5eV to 4.3 eV) and high transmittance (~85% to ~90% in the visible range) [7, 8].

In this work, ITO films with various thicknesses have been employed. Numerous studies have been carried out on the highly transmissive layer of ITO of different thickness. ITO has been sputtered on polished silicon, glass and textured wafer for 10mins, 14mins, 18mins and 22 mins to grow ITO layer of thicknesses 40nm, 60nm, 80nm and 100nm hence named as ITO_40, ITO_60, ITO_80 and ITO_100, discussed in the experimental part of the main article. The thicknesses of the samples are measured by thickness profilometer (DEKTAK-XT, Bruker) to confirm the exact thicknesses of the ITO. Respective transmission and reflection of ITO film of different thickness on glass and wafer have been carried out in Bentham PVE 300. Absorption measurement of the different samples on glass substrates has been carried out in UV-Vis spectrometer. The fractions of reflected, transmitted and absorbed photons in the different ITO layer thicknesses are calculated to quantify the injected photon fraction in the active silicon layer.

It is found that with the increment of ITO thickness on the textured silicon wafer, the reflection minimizes in an almost linear fashion. Bare textured silicon wafer shows a reflection higher than any of ITO coated textured wafer. The integrated total number of photon is found to be $2.1034 \times 10^{17} cm^{-2} s^{-1}$ in AM1.5G from 300nm to 1100nm wavelength, which has been calculated by 'Simpsons 1/3rd' rule.

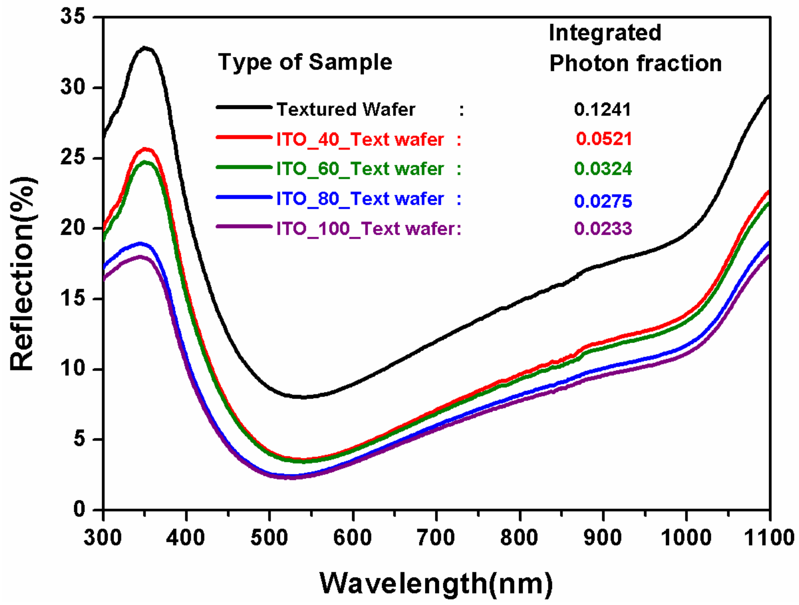

Figure 2: Reflectance graph of ITO coated (Different thickness) textured silicon wafer

In order to quantify the total numbers of photons reflected from the bare textured silicon surface and ITO_40, ITO_60, ITO_80 and ITO_100 coated textured Si wafer surfaces are calculated 'Simpsons 1/3rd' rule. In order to obtain a rational idea about the change in reflected photon, fractional studies of all the reflected photons have been estimated w.r.t. the total no of integrated photons in AM1.5G using equation 3 [9, 10], where $R(\lambda)N_0(\lambda)$ and $N_0(\lambda)$ are the reflected and total photon count respectively within the said wavelength regime . Reflection graph has been downshifted significantly after the coating of ITO on bare textured Si wafer (Figure 2). The integrated reflected photons fraction from the surface of the bare textured Si wafer is 0.1241, whereas for ITO_40, ITO_60, ITO_80 and ITO_100 coated textured Si wafer are 0.0521, 0.0324, 0.0275 and 0.0233 respectively (table 1). With the increment of the thickness of the ITO thin film, reflected photon fraction decreases linearly. According to the equation 2, 80nm ITO should show least reflected photon fraction than 100nm ITO coating. Further decrement in reflection on 100nm ITO coating is due to the parasitic absorption loss by ITO thin film. Absorption loss has been measured and calculated to attain the fair perception from the absorption graph (Figure 3) leading to the selection of optimized thickness of ITO coating on the textured Silicon wafer.

$$n_{ph}^{ref} = Refected\ Photon\ fraction\ (RPF) = n_{ph}^{ref} = \frac{\int_{300nm}^{1100nm} \mathrm{R}\ (\lambda)\mathrm{N}_0(\lambda)\mathrm{d}\ \lambda}{\int_{300nm}^{1100nm} \mathrm{N}_0\ (\lambda)\ \mathrm{d}\ \lambda} \quad (3)$$

Table1: Calculation of photon fraction from reflection, absorption and transmittance of ITO of different thicknesses.

	Type of sample	Measurement taken on Wafer		Measurement taken on Glass		Measurement taken on Glass		
		TRP	FRP	TAP	FAP	TTP	FTP	ITPF
Total Photons (Global) 2.1034×10^{17} $cm^{-2}s^{-1}$	Textured wafer	2.6236×10^{16}	0.124					
	ITO_40	1.0957×10^{16}	0.052	1.1423×10^{14}	5.43E-4	1.8203×10^{17}	0.8655	0.82041
	ITO_60	6.8063×10^{15}	0.032	1.3329×10^{14}	6.34 E-4	1.8423×10^{17}	0.8853	0.8566
	ITO_80	5.7922×10^{15}	0.027	1.4716×10^{14}	7 E-4	1.8800×10^{17}	0.8939	0.8693
	ITO_100	4.9214×10^{15}	0.023	1.5123×10^{14}	7.18 E-4	1.8419×10^{17}	0.8756	0.8552

TP: Total photons in AM1.5(Global) from 300nm to 1100nm; $cm^{-2}s^{-1}$
TRP: Total reflected photons from the top surface from wavelength 300nm to 1100nm; $cm^{-2}s^{-1}$
RPF: Reflected photon fraction (TRP/TP); No unit
TAP: Total absorbed photons in ITO; $cm^{-2}s^{-1}$
FAP: Absorbed photon fraction (TAP/TP); No unit
TTP: Total transmitted photons through ITO layer from 300nm to 1100nm wavelength; $cm^{-2}s^{-1}$
FTP: Fraction of transmitted photons through ITO layer (TTP/TP); No unit
ITPF: Injected total photon fraction {FTP×(1-FRP)}; No unit

Reflection data is not sufficient to comprehend the number of photons absorbed by the silicon layer. There are many other parameters which play a great role on the estimation of the injected photons to the silicon layer. Before reaching the silicon substrate light has to travel through the ITO layer. Due to having very less absorption co-efficient [11], insignificant light gets absorbed by the ITO and some other parasitic absorption takes place.

$$Absorbed\ Photon\ fraction\ (APF) = n_{ph}^{Ab} = \frac{\int_{300nm}^{1100nm} \mathrm{A}\ (\lambda)\mathrm{N}_0(\lambda)\mathrm{d}\ \lambda}{\int_{300nm}^{1100nm} \mathrm{N}_0(\lambda)\mathrm{d}\ \lambda} \quad (4)$$

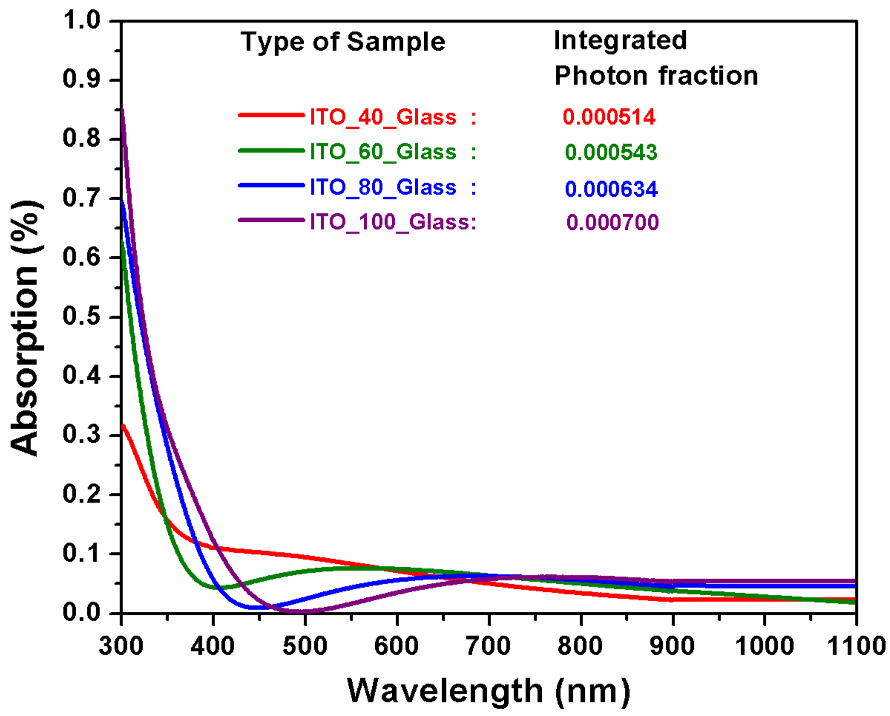

Figure 3: Absorption curve of different thicknesses of ITO coating on glass substrate.

As we go down in table 1, absorption increases with increment of the thickness of the ITO layer, but not very significant in magnitude (figure 3). Absorption by ITO_40, ITO_60, ITO_80 and ITO_100 coated glass substrates were measured in the UV-Vis NIR spectrometer and the integrated photon absorption fractions are 5.43×10^{-4}, 6.34×10^{-4}, 7.0×10^{-4} and 7.18×10^{-4} respectively (equation 4 [9, 10]), which is negligible w.r.t. total number of photons incident from AM1.5G and hence the changes in absorption fraction is neglected during the time of injected photon calculation. $A(\lambda)N_0(\lambda)$ and $N_0(\lambda)$ are absorbed and total photon counts respectively.

It is observed from the transmittance graph (Figure 4) that with the increment in the thickness of the ITO on glass the transmittance graph shows an inverse relation with the absorption. ITO_40 coated glass shows maximum hindrance to UV to mid visible region and better transmittance in the infra-red region, whereas ITO_100nm and ITO_80 show less hindrance in entire wavelength regime. With the increment of the ITO thickness we have been able to shift the transmittance maxima towards lower wavelength.

The obtained data of transmittance has been calculated to comprehend the exact transmittance in the measured wavelength regime. Total transmitted photons have been calculated for ITO_40, ITO_60, ITO_80 and ITO_100 by Simpson's 1/3rd rule, which estimates the exact photon count

that reaches active silicon layer by equation 5 [7, 10], where $T(\lambda)N_0(\lambda)$ and $N_0(\lambda)$ are the transmitted and total photon counts respectively.

$$Transmitted\ Photon\ fraction\ (TPF) = n_{ph}^{tr} = \frac{\int_{300nm}^{1100nm} \mathrm{T}\,(\lambda)\mathrm{N}_0(\lambda)\mathrm{d}\,\lambda}{\int_{300nm}^{1100nm} \mathrm{N}_0\,(\lambda)\,\mathrm{d}\,\lambda} \qquad (5)$$

It is seen that, due the blue shifting of transmittance maxima with the increase of ITO thickness, solar cell active visible spectrum was transmitted through ITO layer with less obstruction. In this comparative study shown in table 1, ITO_80 shows utmost transmittance with photon fraction 0.8939, than ITO_100 (0.8552), ITO_60 (0.8566) and ITO_40 (0.8204) thick ITO.

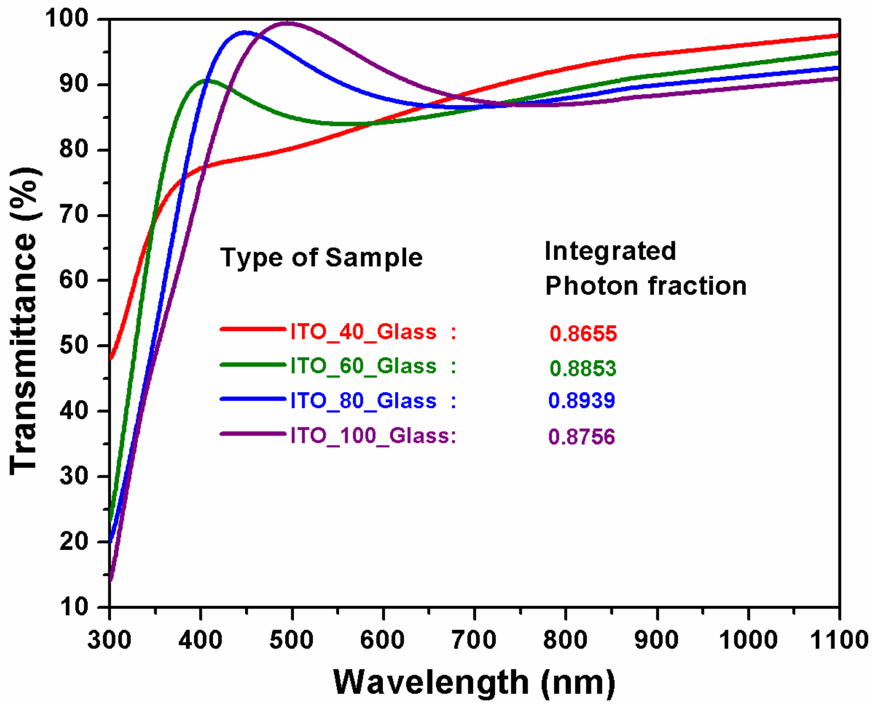

Figure 4: Transmittance curve of different thicknesses of ITO coating on glass substrate.

From the table 1, the injected photon has been calculated, which is finally absorbed by the active silicon solar cell, where many optical phenomenon are involved in its due course of travel, which are i) reflection loss from the surface of the ITO layer ii) parasitic absorption loss at the ITO layer. iii) Reflection loss from the surface of the textured silicon iv) due to not achieving exact quarter wavelength thickness of the ARC i.e. ITO, responsible for destructive interference at the ITO surface.

Taking into account all the constraint during the calculation of the injected photon fraction, the absorbed photon fraction at ITO layer is neglected as its measures non-contributory in consideration. Two significant assumptions in this issue are the transmitted photon fraction and reflected photon fraction. Injected photon fraction (IPF) has been calculated in table 1 following equation 6 [9,10].

$$n_{ph}^{in} = n_{ph}^{tr}\left(1 - n_{ph}^{ref}\right) \quad (6)$$

Where,

$$n_{ph}^{in} = Injected\ Photon\ Fraction\ (IPF)$$

$n_{ph}^{tr} = Transmitted\ Photon\ fraction\ (TPF)$, obtained from equation 5,

and $n_{ph}^{ref} = Reflected\ Photon\ fraction\ (RPF)$, obtained from equation 3.

It is noticeable in the table 1 that, integrated photon fraction has been calculated for all the samples. From the respective optical study and corresponding analysis it has been found that the injected photon fraction is maximum i.e.~ 0.87 for ITO_80 in comparison to ITO_40 (0.8204), ITO_60 (0.8566) and ITO_100 (0.8552), also strongly supports the previously discussed quarter wavelength theory in equation 1 and the intermediate RI theory in equation 2.

In urge to establish the ITO as good TCO, sheet resistance measurement was performed. If transparent oxide (85% transmittance in the visible range from 400nm to 700nm [7, 8]) supports electrical resistivity lower than 5.0 x 10^{-4} Ω - cm (1.44 to 2.08) can be employed as solar potent TCO material in solar cell structure [8]. Resistivity of ITO strongly associated with a few factors, which are as following i) the resistance of ITO films decreases with increase in oxygen vacancy in the lattice network. The oxygen vacancies create free electrons in the films because one oxygen vacancies creates two extra electrons [11]. The increase in the number of oxygen vacancies leads to an increase in carrier density and a consequent decrease in resistance ii) If many free electrons exist in ITO films, the mobility is rapidly decreased by scattering with carriers or with crystal defects [12] iii) Resistivity can increase due to the change in chemical composition or micro-crystal structure of an ITO film [13] iv) Mobility is said to be increased due to enhanced crystallinity of films deposited at higher substrate temperatures. The results discussed in these studies vary significantly from one another and suggest that the optical and electrical attributes significantly depend on thickness manipulation of ITO layer and demand a careful experimental and mathematical data investigation to select optically perfect thickness of

ITO to be used in the solar cell as TCO for reproducible results. In terms of optical and electrical perceptions, 80nm thick ITO (ITO_80) deposited on substrate in our case, shows optimized performance with 87% transmittance and 50 Ω/□ sheet resistance.

3. Characterization of the RGO layers

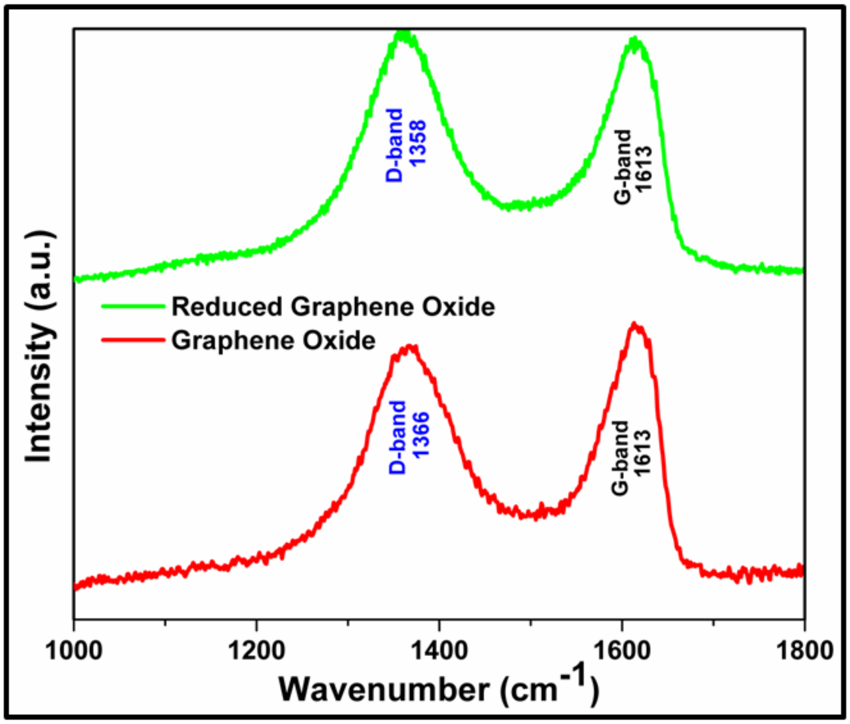

Figure 5: Intensity variation of D and G peaks in Raman spectroscopy of GO and RGO

Raman spectroscopic study (RanishawinVia.) with laser excitation wavelengths of 514 nm (2.41eV) was used to investigate the G'-band as a function of thickness and is presented in figure 5. For the measurement, graphene thin film was coated on glass substrate by spin coating. Each of the de-convoluted Raman spectra shows two peaks in the figure; disorder-induced (D) band and tangential (G) band. G band is usually assigned to the E_{2g} phonon of sp^2carbon atom, while D band is a breathing mode of κ-point phonons of A_{1g} symmetry. Two distinct peaks at ca. 1366cm^{-1} and 1613 cm^{-1} correspond with D and G bands in GO. The shifting and intensity gain in D band (1358 cm^{-1}) indicates that the graphene sheets (RGO) has structural disorder. Intensity ratio of D and G band (I_D/I_G) in RGO is 1.04 and in GO is 0.91. The comparison shows a decrease in the average size of sp^2 domain due to the reduction process [14].

References

1. L. Wei, C. Shuying, *J. Semicond*. **2011**, 32, 013002.

2. M. Gulen , G. Yildirim , S. Bal, A. Varilci, I. Belenli, M. Oz, *J. Mater Sci: Mater Electron*. **2013**, 24, 467.

3. M. A. Green, M. J. Keevers, *Prog in photovoltaics: Res and application*.1995, 3, 189.

4. J. Zhao, M. A. Green, *IEEE Transaction on Electron Devices***1991**, 38, 1925.

5. K. L. Chopra, S. Major, D. K. Pandya, *Thin solid Films*1983, 102, 1.

6. Y. Yang, X. W. Sun, B. J. Chen, C. X. Xu, T. P. Chen, C. Q. Sun, B. K. Tay, Z. Sun, *Thin Solid Films***2006**, 510, 95.

7. H. Kim, J. S. Horwitz, G. Kushto, A. Pique, Z. H. Kafafi. C. M. Gilmore and D. B. Chirsey, *J. Appl. Phys*. **2000**, 88, 6021.

8. H. Ohta, M. Orita, M. Hirano, *Appl. Phys. Lett.***2000**, 76, 2740.

9. A. Nandi, S. Majumdar, S. K. Datta, H. Saha, S. M. Hossain, *J. Mater. Chem. C*2017, 5, 1920.

10. H. Ghosh, S. Mitra, S. Dhar, A. Nandi, S. Majumdar, H. Saha, S. K. Datta, C. Banerjee, *Plasmonics***2017**, 12, 1761.

11. O. Tuna, Y. Selamet, G. Aygun, L. Ozyuzer, *J. Phys. D: Appl. Phys.***2010**, 43, 055402.

12. D. R. Caims, R.P. Witte, D. K. Sparacin, S. M. Sachsman, D. C. Paine, G. P. Crawford,*Appl. Phys. Lett*. **2000**, 76, 1425.

13. S. Ishibashi, Y. Higuchi, Y. Ota, K. Nakamura, *J. Vacuum Sc. & Technol.***1990**, A8, 1399.

14. Y. Zhou, Q. Bao, L. A. L. Tang, Y. Zhong and K. P. Loh, *Chem. Mater.***2009**, 21, 2950.
